# A Multi-Beam Phased Array Receiver Front-End with High Performance Ceramic SiP

**DOI:** 10.3390/mi17010110

**Published:** 2026-01-14

**Authors:** Haifu Zhang, Li-Xin Guo, Shubo Dun, Xiaoming Li, Xiaolong Xu

**Affiliations:** 1 School of Physics, Xidian University, Xi’an 710071, China; zhflpp@163.com; 2 54th Research Institute of CETC, Shijiazhuang 050081, China; 3932100132@139.com (S.D.); adamcti@163.com (X.L.); xuxiaolongphd@163.com (X.X.); 3 School of Physics, University of Electronic Science and Technology of China, Chengdu 611731, China

**Keywords:** phased array, SiP, HTCC, multi-beam, front-end module

## Abstract

This paper presents a compact four-beam dual-polarized phased array with the high performance front-end module based on system-in-package (SiP) technology. By employing high-temperature co-fired ceramic (HTCC) substrates, the proposed design achieves efficient thermal management and high level of integration within a tile-type architecture. The front-end module based on SiP can simultaneously generate four independent beams with switchable left- and right-hand circular polarizations, providing flexible beam control. To verify the proposed method, a Ku-band 256-element phased array receiver with four beams has been designed and experimentally verified using HTCC and SiP process. Operating in 14–14.5 GHz, the proposed low-profile array demonstrates stable radiation characteristics, beam pointing accuracy and excellent beam consistency across the entire frequency range. The measurement results confirm that the SiP-based phased array maintains efficient thermal management, high polarization purity and robust beam-scanning capability, validating its suitability for mobile satellite communication.

## 1. Introduction

Satellite communication (SATCOM) systems are currently undergoing intensive research and development, aiming to deliver low-latency, high-throughput broadband connectivity with seamless global coverage. This is intended to address the stringent requirements of emerging applications, including mobile communications, remote internet access, and integrated 6G space-air-ground-sea networks. The stable operation of SATCOM systems relies on continuous alignment between the main beam of ground terminal antennas and target satellites. However, from a terrestrial perspective, satellite positions exhibit significant dynamic variations, primarily due to the high-speed orbital motion of low Earth orbit (LEO) satellites and the orbital perturbations of geostationary Earth orbit (GEO) satellites. Traditional mechanically steered antennas are plagued by inherent drawbacks, such as bulky configurations, sluggish response speeds, and excessive power consumption, which severely restrict their deployment on lightweight mobile platforms. By contrast, phased array systems enable contactless beam scanning but are confronted with critical technical bottlenecks, including the trade-off among gain, bandwidth, and scanning range, high manufacturing costs, and beam squint effects at high frequencies. Therefore, there is a strong demand for a cost-effective SATCOM phased array antenna system with agile beam-steering capabilities [1,2].

The growing demand has driven advancements in front-end modules for phased array systems [3,4,5]. Compared with III-V compound semiconductor technologies, advanced silicon-based semiconductor processes are widely adopted in phased array designs due to their high cost-effectiveness and strong integration capability. However, phased array receivers implemented using these processes still struggle to satisfy the requirements of multi-user concurrent communication and dual-circular polarization beam switching. Consequently, research efforts have increasingly shifted toward multi-beam phased arrays [6,7,8,9,10,11,12], which are predominantly realized using CMOS technology.

Meanwhile, in dual-polarized and multi-beam systems, the growing number of microwave integrated circuits (ICs) and combiner networks presents considerable challenges in circuit layout and thermal management, making it particularly difficult to realize low-profile dual-polarized multi-beam architectures. Furthermore, the total number of phase-shifting channels is determined by the product of the number of polarization ports and the number of beams [13]. However, for a four-beam dual-polarized configuration, both the integration complexity and the thermal dissipation burden increase significantly, accompanied by a corresponding rise in system cost. Previous designs [13,14,15,16,17,18] often achieved system integration at the expense of increased profile thickness. Some approaches lead to several drawbacks, including an elongated heat dissipation path, a larger array profile, and substantially higher assembly complexity. Therefore, multi-beam phased array design faces critical challenges in achieving low-profile, dual polarization, thermal management, and high integration simultaneously.

In this work, a dual-polarized four-beam radio-frequency (RF) front-end module based on high-temperature co-fired ceramic (HTCC) and system-in-package (SiP) technology is proposed. The design employs HTCC substrates with superior thermal and electrical performance. It effectively addresses the thermal management and electromagnetic compatibility issues of Ku-band tile-type multi-beam RF front-ends, achieving efficient beam synthesis for dual-polarized signals across the four beams. Moreover, the phased array antenna based on HTCC and SiP has a relatively low-profile. The experimental evaluation of the manufactured prototype of a 16 × 16 heat-transfer-efficient, and highly compact phased array antenna system with a measured gain-to-noise temperature (G/T) of 1.1 dB/K at 14.25 GHz is presented. The proposed 256-element phased array receiver is capable of scanning ±45° with stable radiation characteristics, beam pointing accuracy (BPA) and excellent beam consistency. To the authors’ best knowledge, the proposed phased array receiver presents the largest number elements of co-aperture dual-polarized four-beam phased arrays.

The main contributions of this work are: (1) we show experimentally that a front-end module based on HTCC and SiP for four-beam phased array with low cost is feasible; (2) we outline a system-performance based design approach of compact low-prolife phased arrays with dual-polarized four-beam capabilities combining various semiconductor technologies; and (3) we demonstrate a detailed and complete design of the optimized array antenna components including integration with transitions.

## 2. System Design

### 2.1. System Architecture

**Figure 1:**
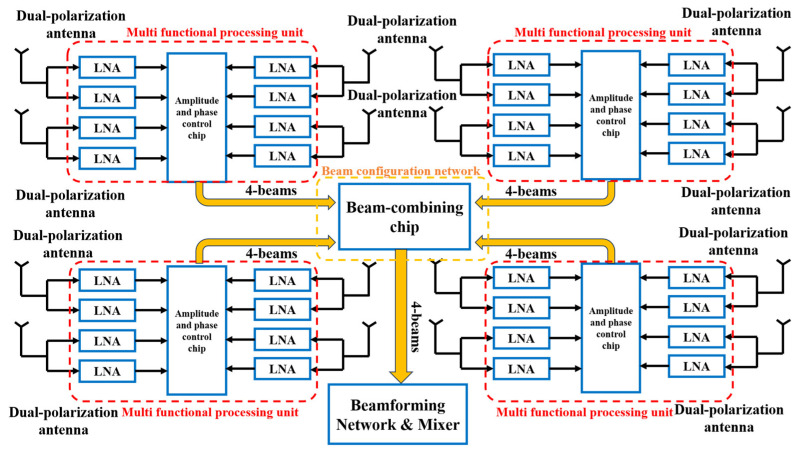
Receiving system block diagram.

The Ku-band four-beam dual-polarized RF front-end consists of a multifunctional processing unit and a beam configuration network. The block diagram of the receiving system is shown in Figure 1. The signals received by the dual-polarized antenna are separated into two polarization ports by an embedded quadrature hybrid and then fed into the multifunctional processing unit. This unit comprises eight low-noise amplifiers and magnitude–phase multifunctional chips, which provide low-noise amplification as well as phase-shifting and attenuation control for the incoming signals.

After processing, the signals from four antenna elements are combined to form a single set of four-beam outputs. Together with the three other sets processed by identical multifunctional units, they are routed to the beam configuration network. The network employs a beam-combining chip to realize the four-beam output of each subarray module. Each subarray module integrates 32 phase-shifting and attenuation channels along with a two-stage beamforming network. Finally, multiple subarray modules are interconnected through the printed circuit board (PCB) motherboard to complete beam configuration and generate intermediate-frequency (IF) outputs.

### 2.2. Structure Profile

**Figure 2:**
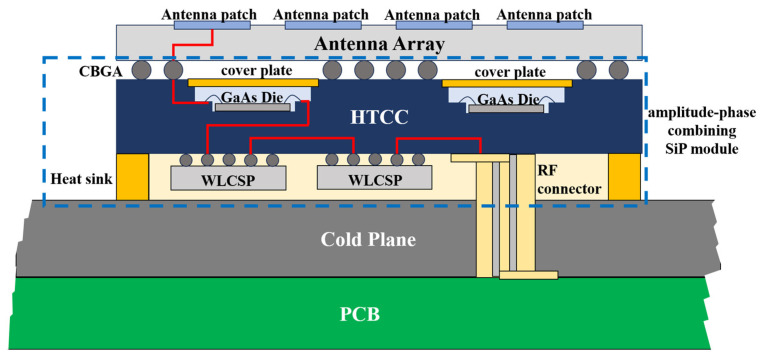
Multi-beam phased array AoP profile and amplitude-phase combining SiP module.

The profile of the proposed AoP module is illustrated in Figure 2. The phased array front-end module is vertically divided into two functional sections, which are mounted on opposite sides of a cold plate. The upper section, located above the cold plate, accommodates the antenna-on-package (AoP) module that integrates the top-layer antenna array with the amplitude–phase combining SiP module. This configuration serves as the fundamental building block of the phased array architecture. Hereinafter, the amplitude–phase combining SiP module is referred to simply as the SiP module.

The lower section beneath the cold plate houses the PCB motherboard, which integrates the RF combining networks along with the power distribution and beam-control networks. The motherboard supplies electrical power and control signals to each AoP sub-module, while simultaneously performing beam-combining functions for all four analog beams. This vertically partitioned architecture ensures efficient thermal management, modular integration, and streamlined signal processing across the phased array system.

**Figure 3:**
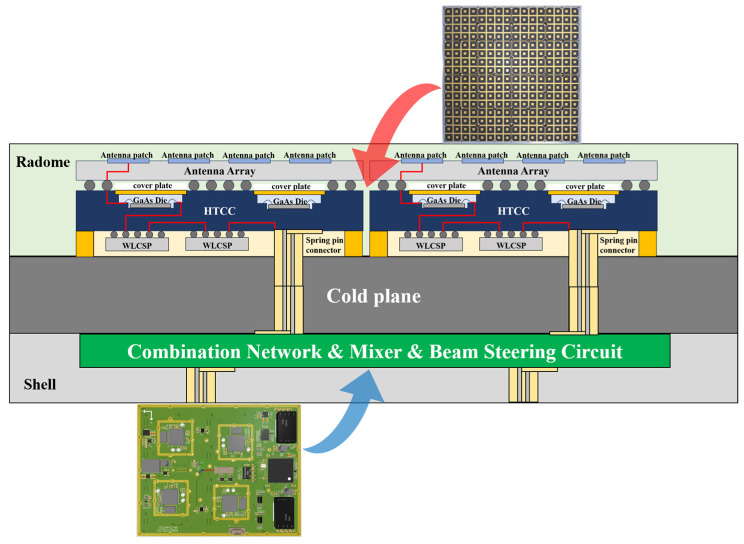
Multi-beam phased array system structure.

Within the AoP module, the SiP module—responsible for amplitude–phase control and beam configuration—is interconnected with the antenna array surface through a ball grid array interface. RF signals pass through the antenna ports and the wafer-level chip-scale package chip mounted on the HTCC substrate. The RF, power, and control connections between the motherboard and AoP modules are established via blind-mate connectors passing through the cold plate. This mechanical and electrical interface is illustrated in Figure 3, showing a 16 × 16-element phased array antenna composed of 16 AoP modules.

## 3. Antenna Patch Design

**Figure 4:**
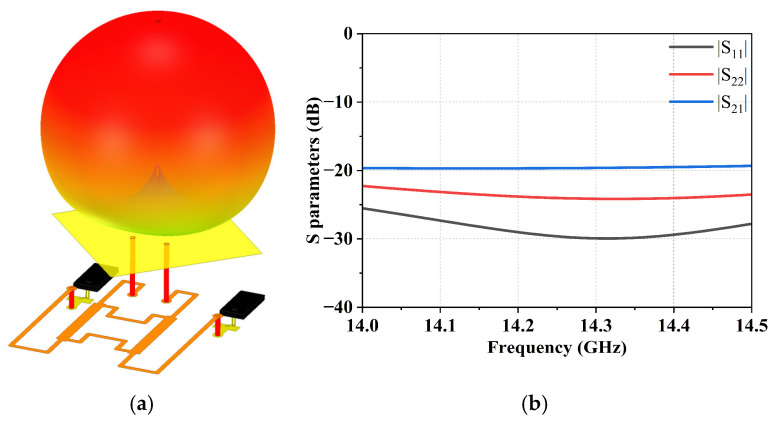
Simulation of the single patch antenna element. (**a**) Simulated model, and (**b**) S parameters.

The dual-polarized antenna element is designed based on a square patch configuration. A 90° phase difference between the two polarization ports is achieved using an internal bridge structure implemented through multilayer dielectric routing. Furthermore, joint optimization with the front-end amplifier’s feed network is performed to minimize feed loss and maximize the sub-array gain. All electromagnetic simulations of the antenna element were performed using ANSYS HFSS 2022 R2. The simulated model and S parameters performance of the single patch antenna element are shown in Figure 4.

**Figure 5:**
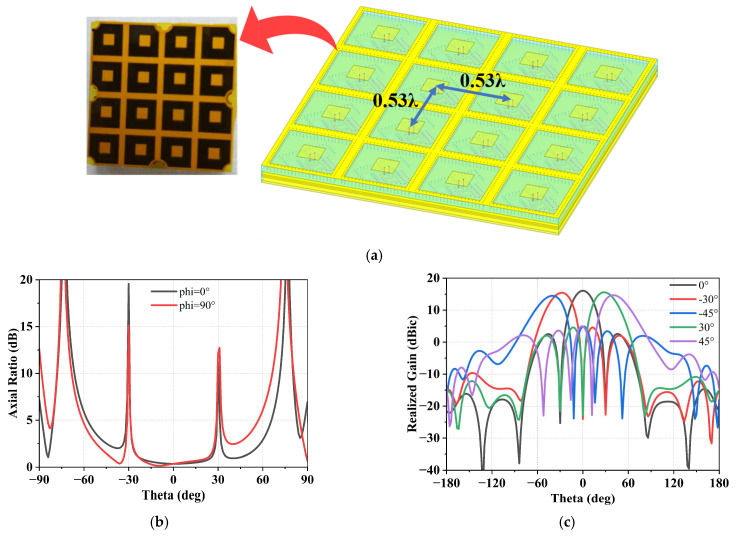
Simulation of the proposed 4 × 4 antenna array. (**a**) Simulated model, (**b**) Axial ratio results at 14.25 GHz, and (**c**) Scanning radiation results at 14.25 GHz.

A 4 × 4 antenna array is employed as the sub-module on the array aperture, which is integrated with the proposed SiP module to form an AoP structure. The designed array supports a beam scanning range of ±45°. When combined with the front-end amplifier and RF switch network, the system enables selectable left-hand and right-hand circular polarizations with negligible RF performance degradation. Figure 5 shows the simulated radiation performance of the proposed 4 × 4 subarray used as a building block of the full phased array. The single-element directivity is 4.58 dBic at 14.25 GHz, and the 4 × 4 subarray achieves a simulated peak directivity of 16.1 dBic. Accordingly, the full 256-element array exhibits a simulated peak directivity of 27.5 dBic, consistent with practical array performance.

## 4. SiP Module Design and Simulation

### 4.1. Electromagnetic Structure Design

The main design challenges of the proposed SiP module lie in achieving high-density in-phase routing and ensuring tolerance control for the RF blind-mate connectors. The in-phase design for signals belonging to the same beam is directly related to the synthesis efficiency of key components within the phased array front-end. When using blind-mate connectors for interconnection, the impacts of manufacturing and assembly tolerances must be carefully evaluated to avoid performance degradation and to preserve an adequate system link-budget margin.

**Figure 6:**
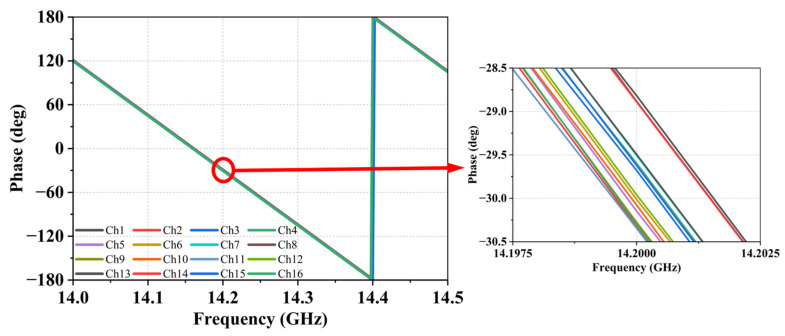
Result of 16 channels in SiP with the same beam phase simulation.

For signals corresponding to the same beam, identical electromagnetic transmission structures and vertical propagation distances are adopted to ensure precise phase consistency. This configuration enables fine phase adjustment on a unified reference plane, thereby achieving equal-phase routing across all signal paths of the same beam at the design level. Full-wave simulation results, shown in Figure 6, demonstrate that the phase deviation among all array elements within the same beam is effectively controlled within 2°, verifying the high precision of the interconnection design.

### 4.2. Structure Heat Dissipation Simulation

**Figure 7:**
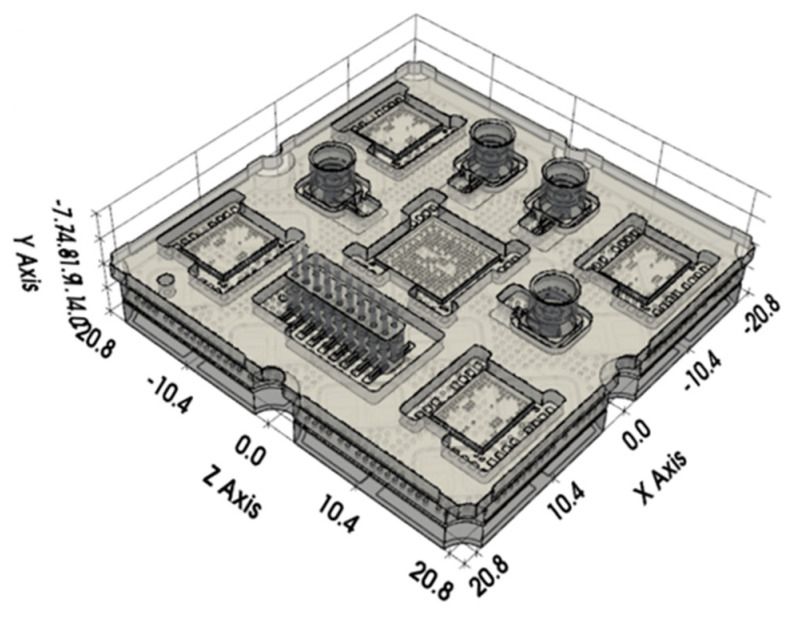
SiP 3D structure diagram.

The SiP structure primarily consists of a HTCC substrate and a bottom-mounted heat sink. The main heat-generating components include the GaAs chip and the wafer-level chip-scale package (WLCSP) multifunctional chip. The heat generated by these active devices is efficiently conducted through the ceramic substrate and subsequently dissipated via the heat sink. The entire SiP assembly is screw-mounted onto the cold plate to ensure effective thermal extraction, as illustrated in the three-dimensional structural diagram shown in Figure 7.

**Figure 8:**
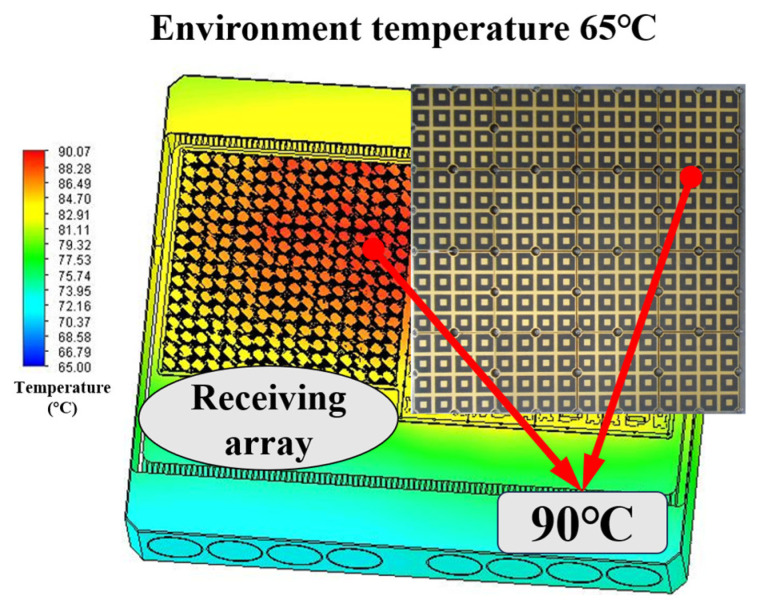
Thermal simulation results.

Under practical operating conditions, when the ambient temperature is 65 °C, the maximum temperature across the 256-element array surface ranges from 56.8 °C to 90.1 °C. The entire structure employs a forced-air cooling mechanism, with heat conduction occurring primarily through the ceramic substrate. Thermal analysis performed using ANSYS Icepak 2022 R2 provides the final steady-state temperature distribution, as illustrated in Figure 8, confirming that the proposed thermal design effectively maintains the SiP module within safe operating limits and ensures reliable performance under high-power conditions. Table 1 displays temperature distribution of the array surface under different operating conditions. As showed as Table 1, within the ambient temperature range of 35 °C to 65 °C, the surface temperature range of the array is 58.6–90.1 °C in full-power operation mode. These results indicate that the proposed array design exhibits excellent heat dissipation characteristics, ensuring reliable operation of the array.

Compared with conventional passive cooling approaches, the proposed forced-air cooling scheme enhances heat dissipation performance by actively augmenting convective heat transfer at the back surface of the array. Routed through dedicated cooling channels integrated beneath the HTCC substrate, the airflow enables efficient extraction of the heat generated by the SiP modules. This design effectively mitigates local hotspots and maintains a more uniform temperature distribution across the entire array, thereby improving thermal stability of the system and ensuring consistent beam performance throughout long-term operation.

## 5. Motherboard Design

**Figure 9:**
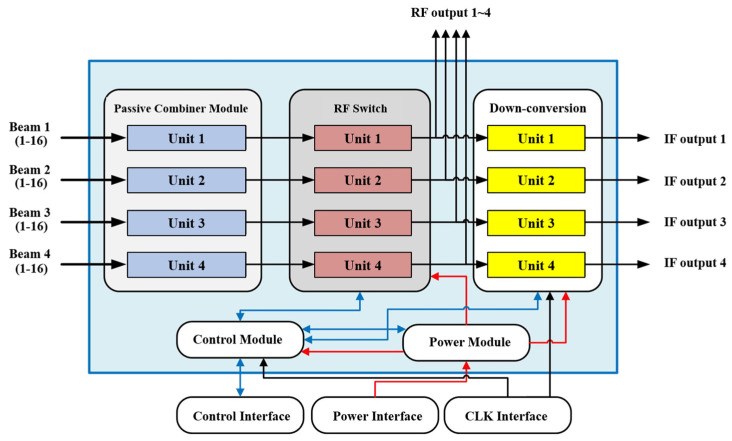
Motherboard functional block diagram.

The motherboard, implemented on a PCB, is primarily responsible for beam signal configuration. Its functions include the feed network for receiving beam signals, frequency conversion between IF and RF, and the distribution of serial peripheral interface control commands to each SiP module. In addition, it integrates part of the power supply circuitry, which provides appropriate voltage levels to various active components. The functional block diagram of the proposed motherboard is illustrated in Figure 9.

**Figure 10:**
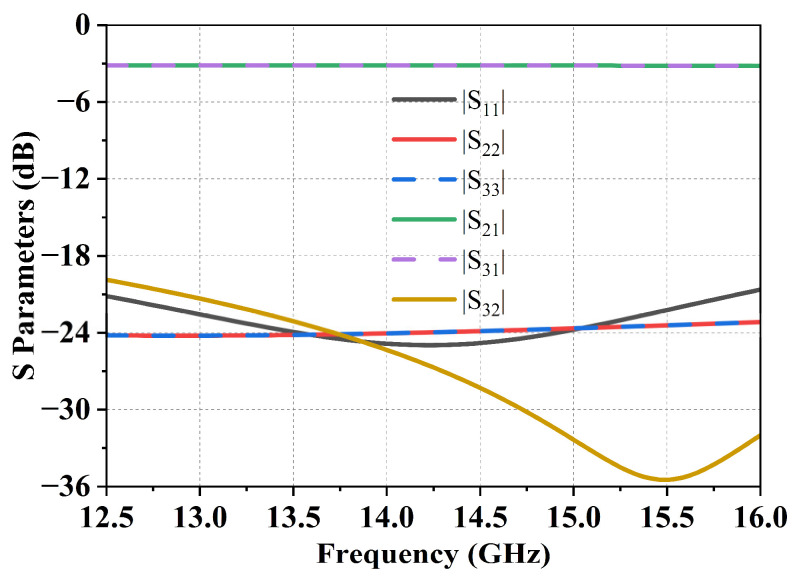
Simulation results of the 2-in-1 combiner.

Each RF combining module aggregates sixteen preceding RF channels into a single RF output. As illustrated in Figure 10, this is accomplished through a cascaded configuration of four embedded two-way combiners. The simulated S-parameter results of the combiner are presented in Figure 10. Within the operating frequency band, the reflection coefficient of all three ports is better than −23 dB, the insertion loss is less than 3.2 dB, and the output port isolation exceeds 24 dB.

**Figure 11:**
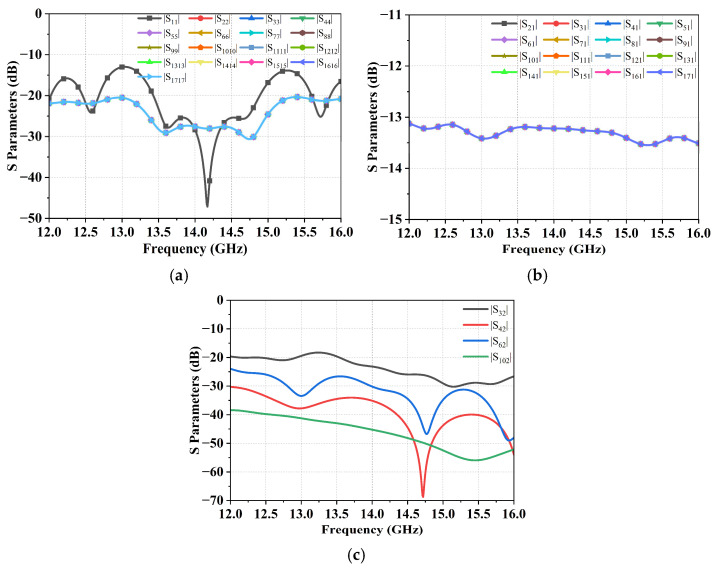
Simulation results of the cascaded 4-in-1 combiner interconnection structure. (**a**) Reflection coefficient, (**b**) Insertion loss, and (**c**) Output isolation.

The full four-stage cascaded 4-in-1 combining module simulation results are shown in Figure 11, demonstrating a reflection coefficient better than −25 dB, an insertion loss below 13.5 dB, and an output isolation greater than 20 dB. These results validate the excellent impedance matching and low-loss characteristics of the designed combiner network.

**Figure 12:**
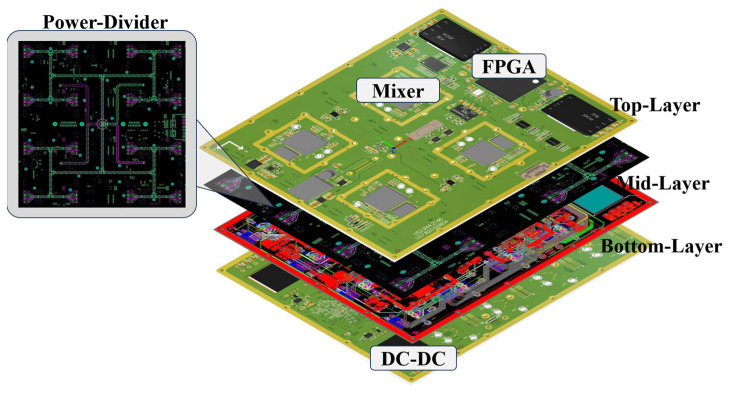
Multi-layer stack-up design.

Based on the cascading verification results, the motherboard for the antenna array surface in the transceiver module was designed using a multilayer hybrid PCB structure. The PCB stack-up configuration and layout planning are depicted in Figure 12, illustrating the spatial distribution of the RF, control, and power layers that ensure compact routing, minimized coupling, and high signal integrity.

## 6. Measurement Results

**Figure 13:**
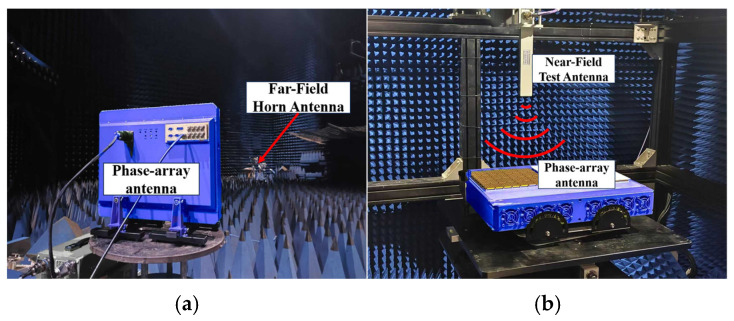
Antenna test environment. (**a**) Far-field test environment, and (**b**) Near-field test environment.

**Figure 14:**
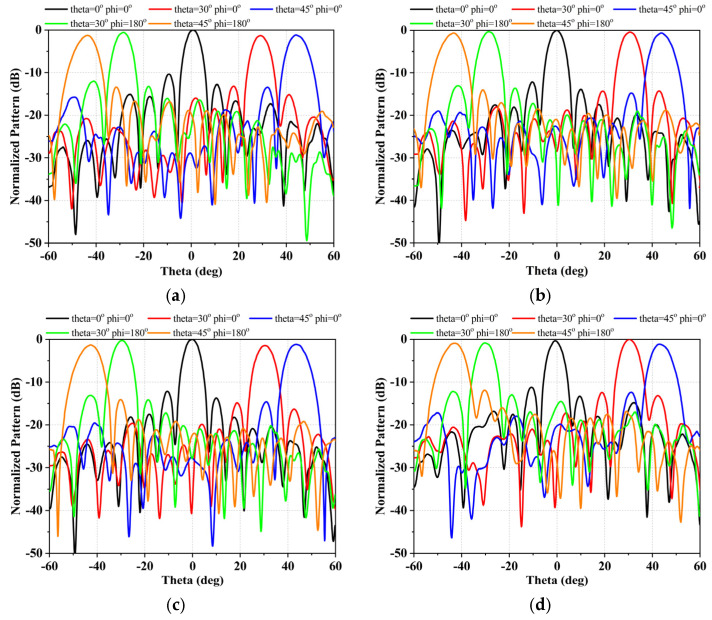
Test results of the radiation pattern of each beam under different elevation angles at 14.25 GHz. (**a**) Beam 1, (**b**) Beam 2, (**c**) Beam 3, and (**d**) Beam 4.

To verify the proposed design, a Ku-band 256-element phased array with four beams has been manufactured and experimentally verified using HTCC and SiP process. The size of the array is 168 mm × 168 mm. Far-field and near-field measurements of the proposed antenna array were carried out in a standard anechoic chamber, as shown in Figure 13. During the experimental evaluation, radiation pattern scans were performed for each of the four beams, as illustrated in Figure 14, which presents the measured radiation patterns under azimuth angles of 0° and 180° with off-axis scanning angles of 0°, 30°, and 45°. 

The G/T of the proposed 256-element phased-array receiver is measured for the four individual beams in an anechoic chamber, where the antenna noise temperature *T*_ant_ is maintained at 295 K. At the center frequency of 14.25 GHz, the measured maximum G/T reaches 1.1 dB/K. At 14.0 GHz and 14.5 GHz, the measured G/T values are 0.7 dB/K and 0.9 dB/K, respectively. Table 2 summarizes and compares the performance of recently reported phased-array receivers. The proposed receiver integrates advanced functionality, including simultaneous four-beam operation with dual circular polarization, within a compact array size. Although some reported designs exhibit wider scan ranges or higher G/T values, system-level characteristics such as profile height, thermal management, and front-end integration are often not fully addressed. In contrast, the proposed design is driven by practical engineering considerations, with an emphasis on low-profile packaging, forced-air cooling, and stable operation under high-power conditions.

The measured results of the design array demonstrate stable radiation characteristics, beam pointing accuracy and excellent beam consistency. The measured beam pointing angle (BPA), 3 dB beamwidth (BW), beam pointing error (BPE) have been achieved in Table 3. The maximum BPE is −0.58 degrees, when the scanning angle of the beam-1 is 45 degrees. From Table 3, at different scanning angles, the BPE of the four beams accounts for less than 8% of the 3 dB beam width. These indicate that the BPA and BPE of the proposed array have good performance. Meanwhile, the amplitudes consistency of the four beams tested is summarized in Table 4. As shown in Table 4, when the array scans 0 degrees, 30 degrees, and 45 degrees, the beam consistency values are 1.71 dB, 0.65 dB, and 1.74 dB, respectively. This measurement results indicate that the proposed antenna array exhibits excellent beam consistency among all four beams. Moreover, stable performance and effective sidelobe suppression are maintained even under large off-axis scanning conditions, confirming the robustness and reliability of the array design in practical multi-beam operation scenarios.

## 7. Conclusions

This paper presents a Ku-band dual-polarized four-beam phased-array front-end module designed using a HTCC process and following the SiP design concept. Through a combination of theoretical design and experimental validation, the proposed front-end module demonstrates low insertion loss and excellent thermal dissipation performance across the operating frequency band.

Notably, it effectively reduces system complexity and overall structural profile, addressing the challenges of high-level integration in multi-beam phased-array systems. Measured results from the array surface verify that the design enables arbitrary polarization configuration, and when integrated with the antenna, the resulting AoP front-end module supports beam scanning up to ±45°. This innovative architecture provides a reliable and scalable solution for next-generation satellite communication and electronic countermeasure systems.

## Figures and Tables

**Table 1:** Temperature distribution of the array surface under different operating conditions.

Environment Temperature (°C)	Device Operating Duration	Temperature Distribution of the Array Surface (°C)
35	Full-power operation	58.6
55	Full-power operation	79.6
65	Full-power operation	90.1

**Table 2:** Comparison of the proposed antenna array with some existing array.

	Frequency (GHz)	Number of Elements	Beam	Polarization	Scan Range (°)	Size (cm^2^)	G/T (dB/K)
[12]	10.7–12.7	16 × 16	2	Dual-Linear	±70	19.5 × 17	5 (*T*_ant_ = 20 K)
[16]	17.7–20.2	32 × 32	1	Dual-Linear	±70	22.4 × 25.2	6.05 (*T*_ant_ = 290 K)
[17]	29	16 × 16	1	Linear	±50	10.4 × 10.4	3.2 (*T*_ant_ = 35 K)
[18]	29.5	8 × 8	4	Linear	±50	4.4 × 4.4	−7 (*T*_ant_ = 290 K)
Prop.	14–14.5	16 × 16	4	Dual-Circular	±45	16.8 × 16.8	1.1 (*T*_ant_ = 295 K)

**Table 3:** The measured beam pointing angle (BPA), 3 dB beamwidth (BW), beam pointing error (BPE) of the array.

Beam Number	Theoretical Calculation of Beam Pointing Angle (°)	Measured Beam Pointing Angle (°)	Measured 3 dB BW (°)	BPE (°)	abs(BPE)/BW (%)
1	0	−0.20	6.08	−0.2	3
2	0	−0.28	6.34	−0.28	4
3	0	−0.25	6.39	−0.25	4
4	0	−0.43	6.34	−0.43	7
1	30	30.30	6.69	0.3	4
2	30	30.56	6.83	0.56	8
3	30	30.26	6.78	0.26	4
4	30	29.62	6.48	−0.38	6
1	45	44.42	8.7	−0.58	7
2	45	44.54	8.69	−0.46	5
3	45	44.80	8.89	−0.2	2
4	45	44.74	8.75	−0.26	3

**Table 4:** The amplitudes consistency of the four beams.

Beam Number	Beam Pointing Angle (°)	Measured Amplitude Level Value in the Maximum Direction (dB)	Maximum Measured Amplitude Level Value (dB)	Minimum Measured Amplitude Level Value (dB)	Beam Consistency (dB)
1	0	−1.71	0	−1.71	1.71
2	0	0			
3	0	−1.31			
4	0	−1.06			
1	30	−1.47	0	−1.47	1.47
2	30	0			
3	30	−0.80			
4	30	−0.33			
1	45	−1.73	0	−1.73	1.73
2	45	0			
3	45	−1.51			
4	45	−0.41			

## Data Availability

All data generated or analyzed during this study are included in this manuscript. There are no additional data or datasets beyond what is presented in the manuscript.
